# Modulation of perceived time caused by stimulus clarity in object recognition

**DOI:** 10.3758/s13414-026-03295-8

**Published:** 2026-06-16

**Authors:** Tetsuto Minami, Shunsuke Terasawa, Yuya Kinzuka, Fumiaki Sato, Shigeki Nakauchi

**Affiliations:** Toyohashi University of Technology, F-403, 1-1 Hibarigaoka, Tenpaku, Toyohashi, Aichi 441-8580 Japan

**Keywords:** Time perception, Object recognition, Object naming

## Abstract

**Abstract:**

This study explored the modulation of perceived time based on subjective visual perception, mainly focusing on object recognition. Unlike physical time, psychological time is subject to various influences, such as attention and stimulus characteristics. We conducted two experiments using images comprising dots to systematically vary stimulus clarity and visual recognizability. Experiment 1 revealed that perceived time was longer when an object was identifiable within the stimulus. Experiment 2 extended this finding by offering three response options related to object recognition and naming. The results showed that named objects further extended the perceived time in participants. These findings suggest that perceived time is influenced by subjective perceptual experiences, such as object recognition and naming, which mediate the effects of objective stimulus attributes on temporal perception.

**Open practices statement:**

The datasets generated and analyzed during the current study are available from the corresponding author upon reasonable request. The experiments were not preregistered.

**Supplementary Information:**

The online version contains supplementary material available at 10.3758/s13414-026-03295-8.

## Introduction

Time perception can be divided into two distinct aspects: physical time, which is objective and measured by clocks, and psychological time, which reflects our subjective experience of duration. Unlike physical time, psychological time is subjective and can vary substantially based on numerous factors. Understanding these variations in temporal experience is crucial for comprehending how humans process and interpret time in their daily lives.

Previous research has identified several important factors that modulate psychological time perception. These include psychological states, such as arousal level (Ono et al., 2007), emotion (Tipples, 2008), and attention allocation (Ulrich et al., 2006); environmental contexts, such as crises (Stetson et al., 2007) or crowded spaces (Shimokawa & Sugimori, 2019); and stimulus characteristics, which are the focus of the present study.

Among these factors, the physical properties of stimuli have been particularly well documented in their effects on time perception. Research on stimulus characteristics has revealed multiple properties that influence temporal perception: larger visual stimuli are perceived as lasting longer than smaller ones (Wehrman et al., 2020), greater numbers of stimuli extend perceived duration (Xuan et al., 2007), and even auditory flutter can systematically alter time perception (Wehrman et al., 2023). These temporal distortions depend on the relationship between successive stimuli rather than their absolute properties, as demonstrated by studies showing that relative, rather than absolute, stimulus size affects perceived duration (Lu et al., 2009; Ono & Kawahara, 2007).

However, investigations into the modulation of perceived time by stimulus clarity and visual recognizability have not yet yielded consistent results. Sasaki and Yamada (2017) used two types of dot patterns as stimuli: one where dots were arranged in a regular grid-like pattern (like a chessboard) and another where the same number of dots was scattered randomly. They found that the regular patterns were perceived to last longer than the random patterns despite both containing the same number of dots. These findings may relate to the impacts of expectation on perception, as demonstrated in studies showing that expected versus unexpected stimuli can modulate temporal perception (Ulrich et al., 2006; Wehrman et al., 2020). The difference in perceived duration between regular and random dot patterns might reflect not only differences in visual complexity but also the degree to which each pattern type meets or violates perceptual expectations, with regular patterns potentially being more predictable and thus processed differently than random configurations. Cardaci et al. (2009) also reported that when multiple paintings were presented and their presentation times were estimated, the more complex the painting, the shorter the presentation time was estimated to be; thus, they reported that complexity affects perceived time. In contrast, Palumbo et al. (2014) reported that stimulus complexity does not affect perceived time. These conflicting findings may reflect differences in how complexity was defined and manipulated across studies. The relationship between stimulus complexity and time perception has been examined using various approaches: Hogan (1975) manipulated the number of interior angles in line drawings, Cantor and Thomas (1977) varied the area and perimeter of geometric patterns, and Folta-Schoofs et al. (2014) found that perceptual complexity, rather than emotional valence or arousal, accounted for temporal overproduction effects. Importantly, Palumbo et al. (2014) proposed that complexity affects subjective duration only when visual complexity has semantic content and engages participants in associative and cognitive processes. This distinction between the perceptual complexity of abstract patterns and the semantic complexity of meaningful stimuli may explain the inconsistent results in the literature (Donderi, 2006).

In the present study, our approach differs from these previous studies in both the nature of our manipulation and our theoretical focus. While previous research primarily manipulated objective stimulus properties – such as spatial configuration (regular vs. random arrangement; Sasaki & Yamada, 2017), semantic richness (complexity of meaningful paintings; Cardaci et al., 2009), or computational complexity (edge density and compression ratios in abstract patterns; Palumbo et al., 2014) – we manipulate stimulus clarity to examine how subjective recognition success affects temporal perception.

Specifically, our Dots method manipulation degrades recognizable objects by varying the deformation parameter (g-value) while keeping basic spatial properties relatively constant (same number of dots, similar overall configuration). This degradation creates a continuum of visual recognizability: the same underlying object might appear as an unrecognizable dot pattern, an unclear shape suggesting the presence of something, or a clearly identifiable named object, depending on the degree of stimulus degradation and the observer’s perceptual success on a given trial. This approach allows us to examine whether subjective perceptual experiences, whether an observer successfully recognizes an object, modulate perceived duration, independent of objective stimulus complexity.

This distinction is theoretically important: if time perception depends on objective stimulus properties (as suggested by most previous work), then stimuli with matched physical characteristics should produce equivalent temporal effects. However, if time perception depends on subjective processing success (as we hypothesize), then the same physical stimulus should produce different temporal effects depending on whether recognition succeeds or fails. Our use of subjective reports (visibility ratings, naming ability) as the primary independent variable tests this latter hypothesis.

To test this hypothesis, we adopted a subjective approach that focuses on visual recognizability rather than objective complexity measures. In conventional studies, stimuli are classified according to objective indices, and the data are compared for each classification. However, subjective evaluations of stimuli are not always consistent with objective indices due to individual differences. Therefore, we measured subjective perceptual experiences (whether participants successfully recognized and named objects) rather than relying solely on objective stimulus properties (g-values) as proxies for perceptual outcomes. While objective properties set the conditions for perception, the actual perceptual experience, whether recognition succeeds or fails, mediates the influence on time perception. This study investigated whether subjective success in object recognition, ranging from complete failure to detect any object, through uncertain detection, to successful identification and naming, causes temporal modulation. Our focus on visibility is based on the hypothesis that the amount of brain activity might change and modulate our perception of time owing to differences in processing, such as feature extraction and semantic processing, depending on how people perceive an object. In the coding efficiency framework, one of the models of temporal perception, the perceived time depends on the amount of energy required to encode the stimulus, and it is thought that stimuli that cause stronger neural responses are perceived for a longer time (Eagleman & Pariyadath, 2009). Consequently, if processing differs depending on how a stimulus is perceived, temporal perceptual modulation is expected to occur.

We hypothesized that successful object recognition would modulate perceived duration, with recognized objects being perceived as lasting longer than unrecognized stimuli. Furthermore, we predicted that deeper levels of processing, specifically semantic recognition (the ability to name the recognized object), would lead to even longer perceived durations compared to mere object detection without identification.

In the present study, we conducted an experiment to clarify whether perceptual time modulation occurs depending on the subjective degree of perception for a given stimulus. To investigate the effects of subjective perception, a method that controls the degree of perception without changing the physical characteristics of stimuli is vital. Therefore, we used stimuli created using the Dots method (Moca et al., 2011), which can generate an image that reproduces the contours of the original image by changing the sequence of points in a grid. By changing the gravitational constant g, the appearance of the object in the stimulus (similarity to the original image) can be manipulated without significantly changing image statistics such as luminance and contrast. In addition, we operationally defined object recognition as the participant’s conscious detection of a meaningful object within the stimulus, measured through explicit reports of (a) whether they perceived any object and (b) whether they could name the perceived object. Semantic recognition refers specifically to successful identification and naming. In Experiment 1, we manipulated the clarity of the presence of objects in the stimuli and investigated whether the modulation of perception time was affected by whether an object was visible or not. This experiment aimed to clarify whether modulation of perceived time occurs through object recognition. In Experiment 2, we also examined whether the mere perception of an object causes the modulation of perceived time or whether this occurs only when object naming is possible.

## Experiment 1

### Materials and methods

#### Participants

Twenty-five students (24 male participants and one female participant; mean age 22.0 years, standard deviation 0.9 years) with normal or corrected-to-normal vision participated in the experiment. In designing this study, we did not conduct an a priori power analysis to determine the sample size. Instead, we used a convenience sample of 25 participants based on the availability of participants and practical considerations at our university. To evaluate the adequacy of our sample size, we conducted a sensitivity power analysis following Lakens (2022), which determines the minimum effect size detectable given our sample size, alpha level (0.05), and power (0.80). This analysis revealed that our design was capable of detecting effect sizes of Cohen’s *d* ≥ 0.58, which is well below the observed effect size for the primary visibility effect on point of subjective equality (PSE) (*d* = 0.94). This demonstrates that our sample size was adequate for detecting meaningful effects. All participants received a full explanation of the experiment and task and subsequently agreed to participate. This experiment was conducted with the approval of the “Experiments on Human Subjects” Review Committee of the Safety and Health Committee of the Toyohashi University of Technology.

#### Stimuli

**Fig. 1:**
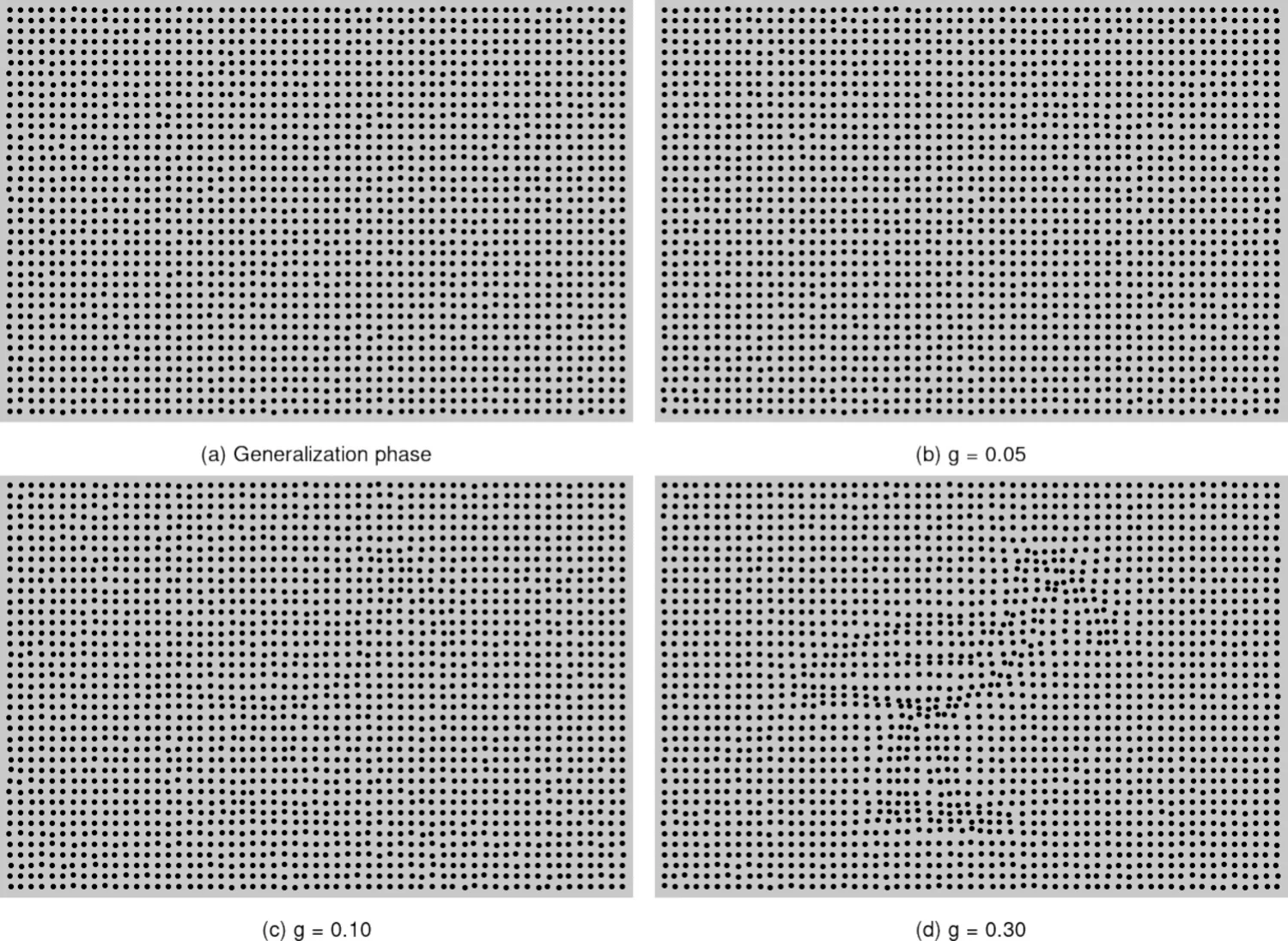
Examples of stimuli used in the experiment. (**a**) The stimuli used in the generalization phase. (**b**)–(**d**) The stimuli used in the bisection task (b: *g* = 0.05, c: *g* = 0.10, d: *g* = 0.30)

The experiments involved stimuli composed of dots created using the Dots method (Moca et al., 2011). In Experiment 1, three patterns (gravitational constant, *g* = 0.05, 0.10, and 0.30) were selected for each image from the images used in a previous study (Moca et al., 2011). The *g*-parameter in the Dots method (Moca et al., 2011) represents the degree of lattice deformation from a regular grid pattern. A *g*-value of 0 indicates a perfect grid arrangement, while increasing values (e.g., 0.30) indicate greater deformation, making the embedded object more recognizable. This parameter effectively controls the visual clarity of the stimulus. The elastic constant K and distance D were assigned the values of 10 and 5, respectively. The three *g* values (0.05, 0.10, and 0.30) were selected based on pilot testing (*n* = 10) that showed these levels produced distinguishable differences in object recognition rates (approximately 20%, 50%, and 80% recognition rates, respectively). A total of 150 images were used in the experiment, consisting of 50 images with each of the three *g* levels. In the generalization phase described below, one image with *g* = 0.0 and random noise was used. The stimulus size was 8.93° × 5.96° at a viewing distance of 96 cm. The stimulus dots were black (RGB 0,0,0) and the background was gray (RGB 200,200,200) (Fig. 1).

For the generalization phase, we intentionally used a stimulus with *g* = 0.0 (perfect grid) with random noise as a control condition. This choice served a specific purpose: the generalization phase aims to establish temporal reference points without any influence from object recognition processes. Using a stimulus with no possibility of object recognition allows participants to focus solely on temporal judgment during this training phase.

The minimum (0.6 s) and maximum (1.4 s) durations were selected based on established temporal bisection paradigms (e.g., Allan & Gibbon, 1991). These durations fall within the sub-second to second range where temporal processing is most relevant for object recognition (Lewis & Miall, 2003). The 0.6-s minimum ensures sufficient time for object recognition processes to occur, while the 1.4-s maximum remains within the psychological present, avoiding potential confounds from explicit counting strategies that might emerge with longer durations. Five test durations (0.6, 0.8, 1.0, 1.2, and 1.4 s) were spaced in equal arithmetic steps.

The presentation time was determined by randomly dividing the 50 stimuli into five sessions for each participant. To examine the specific influence of *g* on the perceived duration of each stimulus separately from the influence of *g*, we ensured that all three patterns of *g* for each stimulus had the same presentation duration. In addition, each stimulus was presented only once to prevent a secondary effect in the degree of perception from multiple repeated presentations.

#### Procedure

This experiment was conducted in a dimly lit room with a desk illumination level of 32.9 lx. The experimental stimuli were controlled using MATLAB 2019b (MathWorks, Natick, MA, USA) and were presented on a monitor (EIZO CS2731-BK ColorEdge, resolution: 2,560 × 1,440 pixels, EIZO, Japan). MilliKey (LabHackers, Halifax) was used to collect the responses. In the generalization phase, participants were first trained to recognize two standard durations: a short duration (0.6 s) and a long duration (1.4 s). During this training, a visual stimulus (a dot pattern) was presented for either 0.6 s or 1.4 s, and participants received feedback about whether their temporal judgments were correct.

Once participants could reliably distinguish these two standard durations, they proceeded to the bisection phase. In this phase, participants were presented with dot pattern stimuli for various durations (0.6, 0.8, 1.0, 1.2, or 1.4 s) and had to judge whether the presentation duration was more similar to the short (0.6 s) or long (1.4 s) standard duration they had learned (Church & Deluty, 1977; Wearden, 1991; Wearden & Ferrara, 1995) and a visibility evaluation task in which they responded on whether they could perceive an object. The order of the bisection task and visibility evaluation task using a visual analogue scale (VAS) was counterbalanced across participants: half performed the temporal judgment first followed by the VAS rating, while the other half completed these tasks in reverse order.

##### Generalization phase

**Fig. 2:**
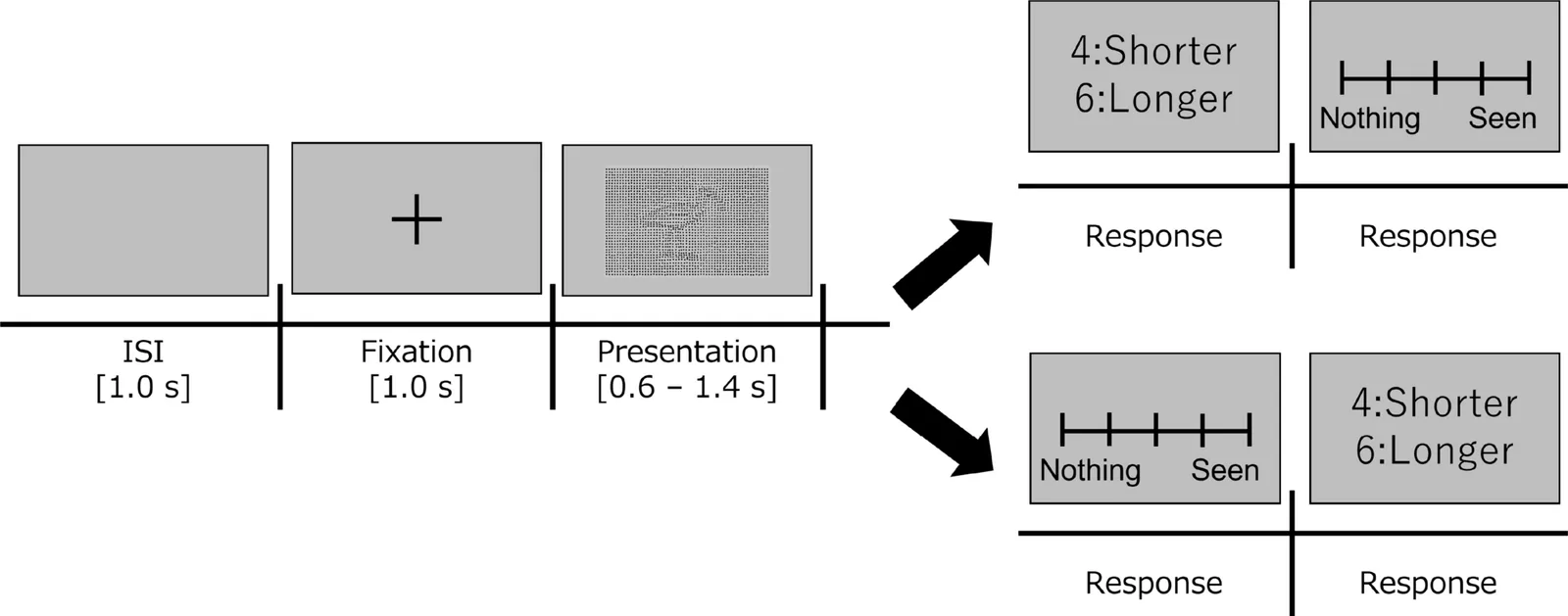
Experimental protocol for one trial in the bisection task. In the bisection task, the presentation durations were 0.6, 0.8, 1.0, 1.2, and 1.4 s, and the participants were presented in a random order. The participants responded by using the 2AFC to indicate whether they felt that the presentation time was closer to the shortest (0.6 s) or longest (1.4 s) time that they had learned in the generalization phase. The visibility evaluation task was conducted in the same way as the generalization phase

The generalization phase was designed to familiarize the participants with the shortest (0.6 s) and longest (1.4 s) durations. Figure 2 illustrates the flow of one trial. A fixation cross was displayed at the center of the screen during the inter-stimulus interval (1.0 s) and the initial fixation period (1.0 s). The fixation cross was removed immediately before stimulus onset to ensure unobstructed viewing of the dot pattern stimuli. The stimulus was then presented for a minimum duration of 0.6 s or a maximum duration of 1.4 s. Participants then performed the bisection and visibility evaluation tasks. In the bisection task, participants responded on whether the stimulus was presented for the shortest (0.6 s) or longest (1.4 s) duration by providing a two-alternative forced-choice (2AFC) response. Feedback was given by displaying an “O” on the screen after the correct response or an “X” if the response was incorrect. The response for whether something was perceived was “something was perceived” if it was to the right of the center, and “nothing was perceived” if it was to the left of the center. The selection toward each end of the VAS represented higher confidence in the response and relatively lower confidence toward the center. For example, the further to the right, the more confidently the participants recognized something, and slightly to the left of the center, the less confident they were about seeing anything. The generalization phase was terminated after six consecutive correct responses to the bisection task. If the participant made even one mistake in the bisection task, the order of presentation times was rearranged randomly, and the task was continued.

##### Bisection phase

In the bisection phase, the bisection and visibility evaluation tasks were performed, and the data were saved. The experimental procedure was the same as that used in the generalization phase, except that no feedback was provided. In the bisection task, the presentation durations were 0.6, 0.8, 1.0, 1.2, and 1.4 s, presented in a random order. Participants engaged in a 2AFC task to indicate whether they felt that the presentation time was closer to the shortest (0.6 s) or longest (1.4 s) duration that they had learned in the generalization phase. The visibility evaluation task was conducted in the same manner as in the generalization phase. The number of trials was 150, consisting of five time durations, three *g* conditions, and ten repetitions, divided into five sessions. The participants were allowed to take breaks between sessions, and the generalization phase was conducted at the beginning of each session. Stimuli were presented in a random order to each participant.

#### Analysis

We conducted two complementary sets of analyses to address our research questions. First, we examined how objective stimulus properties (*g*-values) influenced temporal judgments to confirm that our stimulus manipulation successfully modulated subjective perception. These analyses by *g*-value provide a manipulation check and allow comparison with previous studies using similar stimuli. Second, and central to our research question, we examined how subjective perceptual experiences (VAS visibility ratings) influenced temporal judgments. This approach allows us to assess whether individual differences in perception, even for physically identical stimuli, modulate perceived duration, thereby isolating the role of subjective experience from objective stimulus properties.

For each participant, the response data were analyzed and fitted with psychometric functions using the “quickpsy” R package (Linares & López-Moliner, 2016). We fitted cumulative normal functions (probit model: Ψ(x) = Φ((x—μ)/σ)) to response data, with functions constrained to asymptote at 0 and 1 (no guess/lapse rate estimation). Model fit quality was evaluated using bootstrap deviance statistics (1,000 iterations). For Experiment 1 (75 fits), mean deviance was 8.7 (*SD* = 10.2, mean *p* = 0.83); all fits were acceptable (all *p* >.05). For Experiment 2 (60 fits), mean deviance was 8.57 (*SD* = 12.52, mean *p* = 0.76); three participants showed one poor fit each but were retained as they performed above chance. High mean *p*-values (>.75) indicate good model fit quality. The x-axis was set to the time condition and the y-axis to the percentage of “long” responses for each condition. In the visibility analysis, stimuli were classified according to whether the participants responded “visible” or “invisible” to a certain stimulus. The visibility criterion was based on the VAS response using a 101-point scale ranging from 0 to 1 in 0.01 increments, where 0.5 or higher was considered “perceivable” and less than 0.5 was considered “imperceivable.” In the analysis of confidence in response, stimuli were classified according to whether the response was “confident” or “not confident” based on the visibility response. The confidence criterion was defined as “confident” if the VAS response was less than 0.25 or greater than 0.75 and “not confident” otherwise. The just noticeable difference (JND) and point of subjective equality (PSE) were calculated for each data set, and repeated-measures analysis of variance (ANOVA) and *t*-tests were conducted. For all repeated-measures ANOVAs, Greenhouse–Geisser corrections were applied when sphericity assumptions were violated. The Holm method was used for multiple comparisons, and the significance level was set at α = 0.05. In all figures showing violin plots with PSE and JND data, dark dots represent means and error bars represent 95% confidence intervals.

To examine whether subjective perceptual experiences mediate the relationship between stimulus properties and temporal perception, we conducted trial-level mediation analysis using mixed-effects models. Path a (predicting VAS confidence from *g*-values) used linear mixed-effects regression (lmer). Paths b, c, and c' (predicting binary temporal responses) used generalized linear mixed-effects logistic regression (glmer with binomial family), as appropriate for binary outcomes, with stimulus duration included as a covariate to control for its effect on temporal judgments. All models included random intercepts for participants. Indirect effects were calculated as the product of path coefficients (a × b), with 95% bootstrap confidence intervals computed using 1,000 iterations with participant-level resampling.

### Results

#### Modulation of perceived time by parameter g

**Fig. 3:**
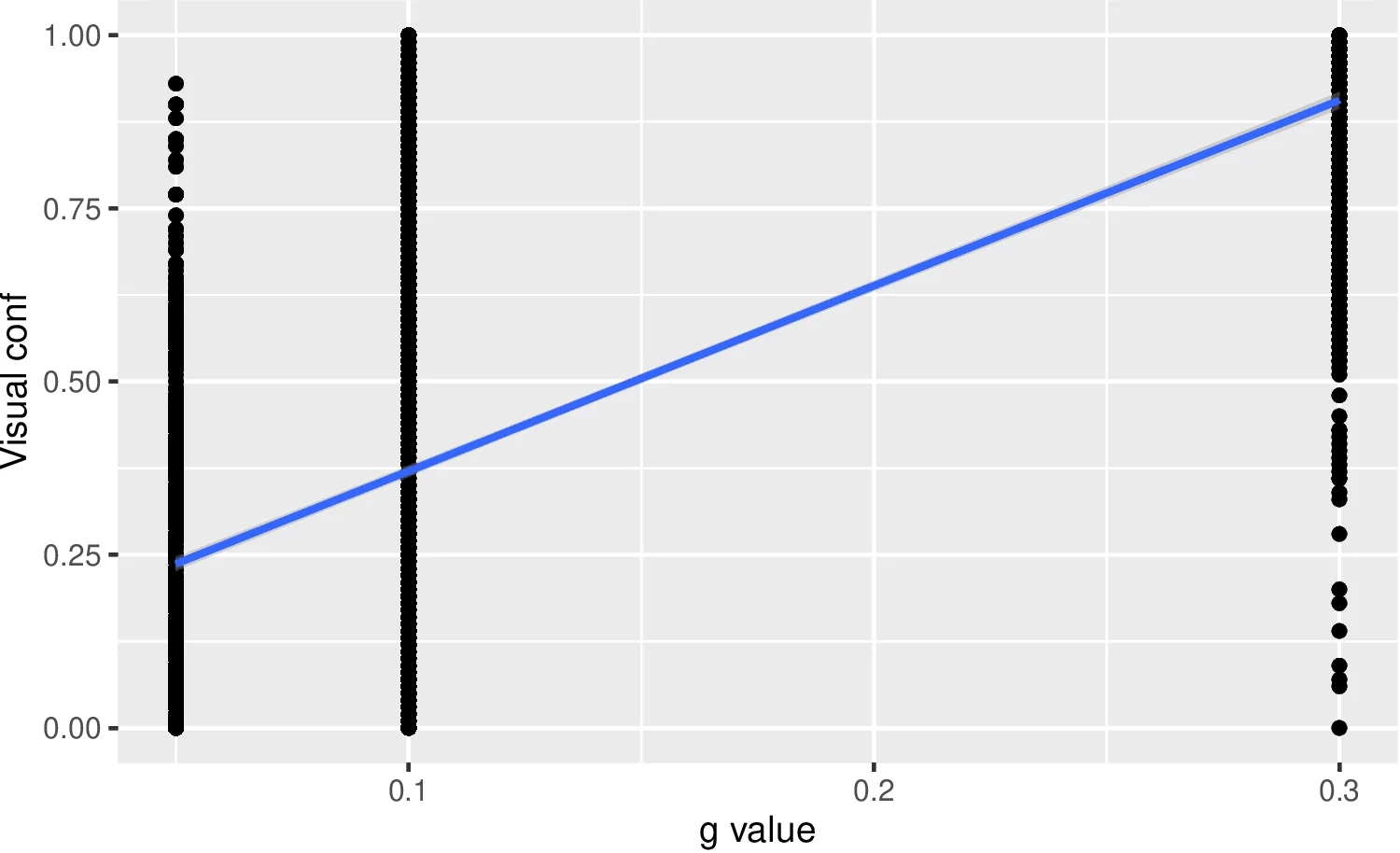
Correlation between *g* value and visual analogue scale responses

We first examined the correlation between *g* values and VAS confidence ratings on a trial-by-trial basis (Fig. 3). We computed Spearman’s rank correlation, which does not assume a linear relationship. The correlation was rs = 0.78, indicating a strong monotonic relationship between stimulus properties and subjective visibility judgments.

**Fig. 4:**
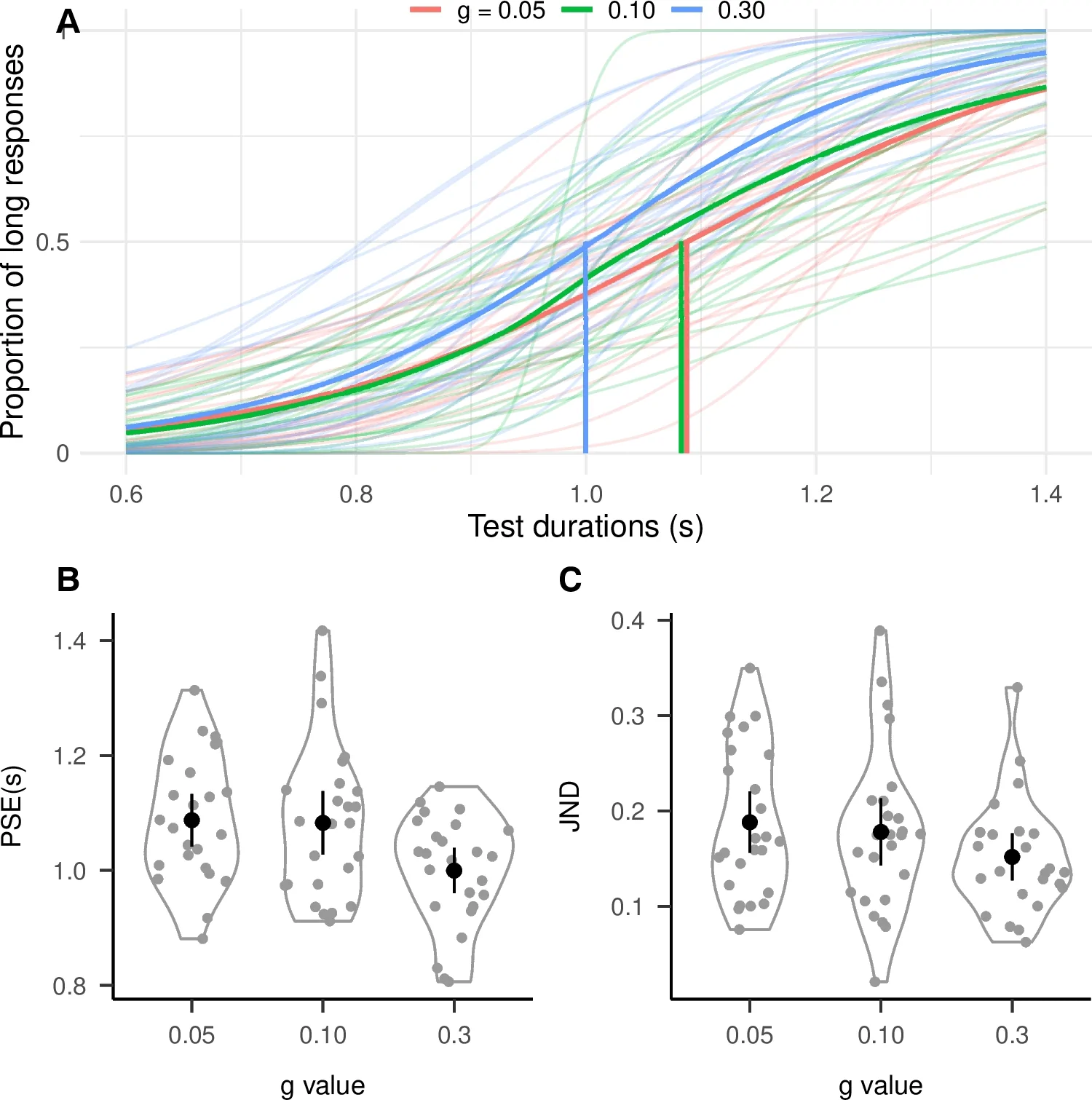
(**A**) Psychometric functions for each *g*. Light colors represent individuals, dark colors represent group levels, and vertical lines indicate the average point of subjective equality (PSE). (**B**) PSE value per *g*. (**C**) Just noticeable difference (JND) value per *g*. As the *g* value increases, PSE decreases. This means that time is perceived to be longer

Figure 4 presents the psychometric functions and parameter estimates (PSE and JND) for each *g*-value condition. As the *g*-value increased from 0.05 to 0.30, the psychometric functions shifted leftward (Fig. 4A), indicating longer perceived durations for stimuli with higher visibility. This effect is quantified by the systematic decrease in PSE values with increasing *g*-values (Fig. 4B), while JND values showed only minimal variation (Fig. 4C).

Repeated-measures ANOVA was performed on the PSE and JND values. The factors included one factor and three levels of parameter *g*. The analysis showed a significant main effect for PSE and JND (PSE: *F*(1*.*89*,* 45*.*26) = 6*.*79, *MSE* = 0*.*01, *p* = *.*003, ${\widehat{\eta }}_{G}^{2}$= *.*115, JND: *F*(1*.*94*,* 46*.*55) = 3*.*54, *MSE* = 0*.*00, *p* = *.*038, ${\widehat{\eta }}_{G}^{2}$= *.*042). No significant differences were found between conditions in JND, but a significant difference was found between conditions in PSE (*g* = 0.05 and *g* = 0.30: *t*(24) = 3.74, *p*_Holm(3)_ = *.*003) and *g* = 0.10 and *g* = 0.30: *t*(24) = 2*.*85, *p*_Holm(3)_ = *.*018). In other words, the participants perceived the stimuli that were easy to recognize for a longer period of time than those for which it was difficult to recognize the object.

#### Modulation of perceived time with and without visibility

**Fig. 5:**
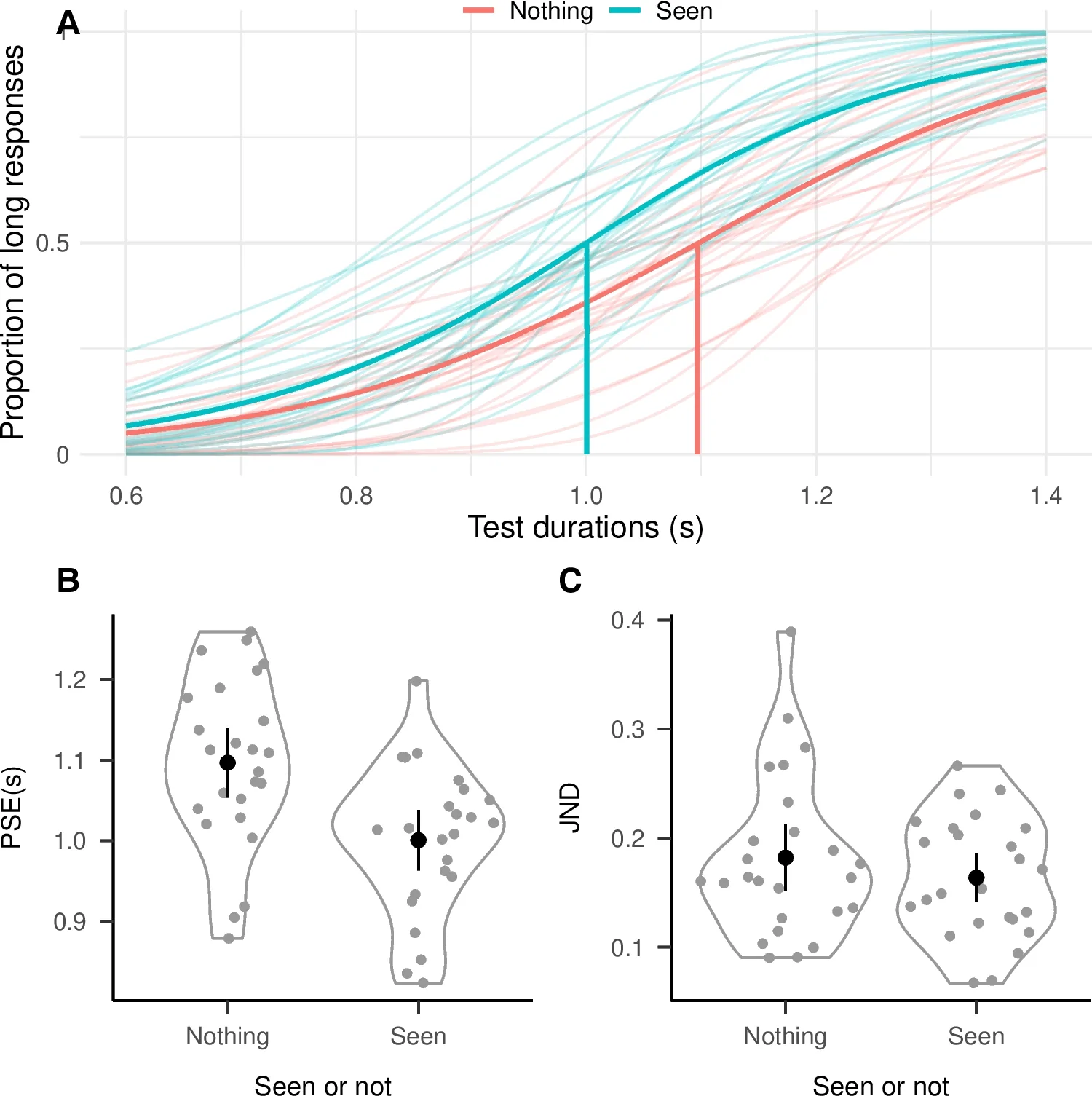
(**A**) Psychometric functions for each visibility. Light colors represent individuals, dark colors represent group levels, and vertical lines indicate the average point of subjective equality (PSE). (**B**) PSE value. (**C**) Just noticeable difference (JND) value. The PSE was smaller for those who responded that they “saw” an object in the dot stimulus. This means that time is perceived to be longer

Figure 5A shows the results of fitting the psychometric curves to the stimuli classified according to visibility. The horizontal axis shows the stimulus presentation time, and the vertical axis shows the percentage of respondents who answered that the presented stimulus was longer than the standard time. Figures 5B and C show the results of PSE and JND for each condition.

The analysis showed a significant difference for PSE in visibility (*M*_*D*_ = 0*.*10, 95% CI [0*.*05*,* 0*.*14], *t*(24) = 4*.*70, *p* < *.*001, *d* = 0.94) but not for JND (*M*_*D*_ = 0*.*02, 95% CI [0*.*00*,* 0*.*04],* t*(24) = 1*.*74, *p* = *.*095, *d* = 0.35). This pattern indicates that participants perceived stimuli to last longer when they could detect an object without any significant change in the precision of their temporal judgments.

This 100-ms difference represents approximately 10% of the mean stimulus duration (1,000 ms), a substantial perceptual distortion. To contextualize this magnitude, the observed difference is approximately 60% of the mean JND observed in our study (mean JND ≈ 170 ms across visibility conditions; see Table S1b in the Online Supplementary Material), indicating that the effect of object recognition on perceived duration is of substantial magnitude and readily perceptible. The large effect size (Cohen’s *d* = 0.94) further confirms that object recognition produces robust and reliable changes in temporal perception.

#### Modulation of perceived time with and without confidence

**Fig. 6:**
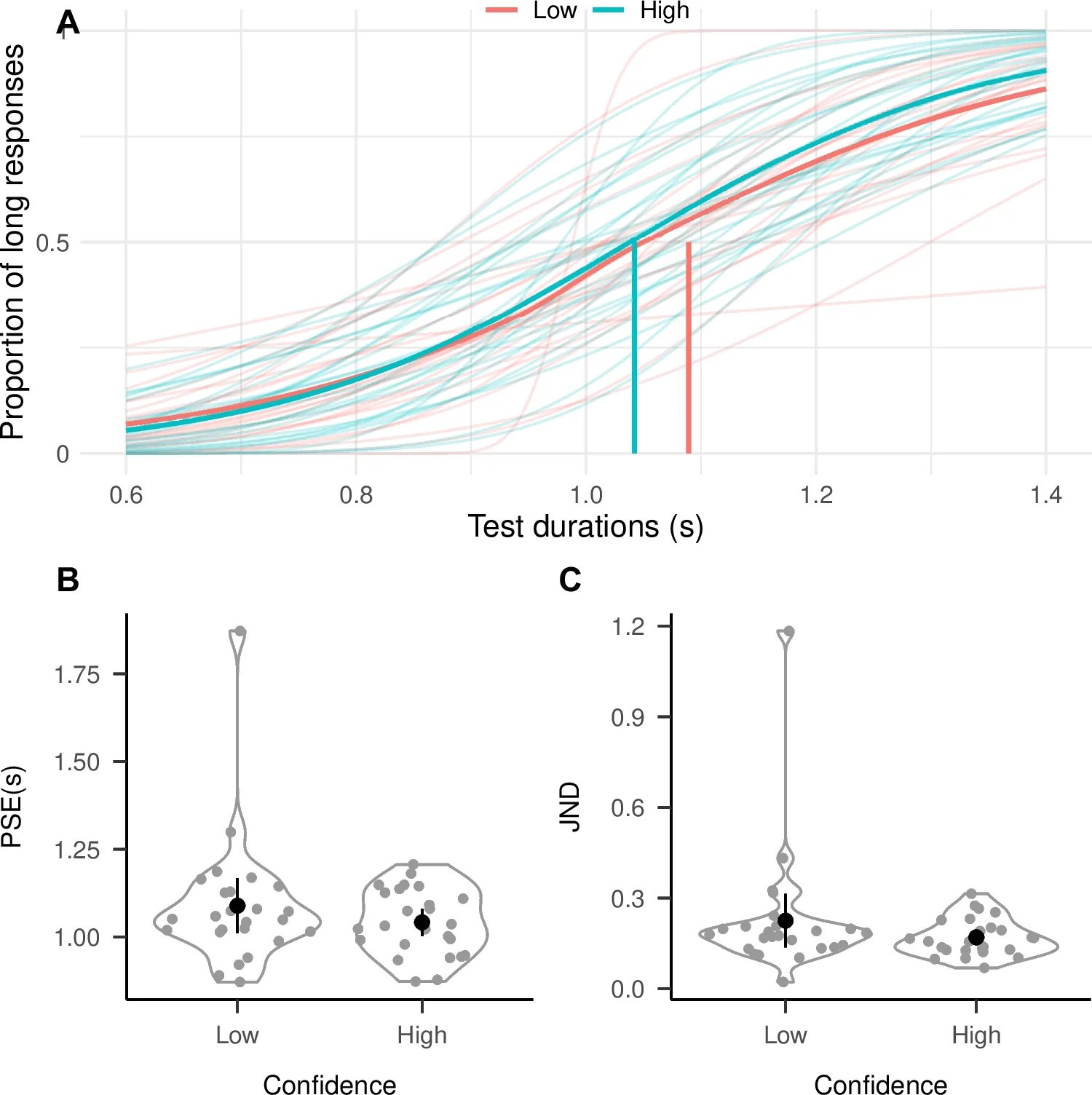
(**A**) Psychometric functions for confidence. Light colors represent individuals, dark colors represent group levels, and vertical lines indicate the average point of subjective equality (PSE). (**B**) PSE value. (**C**) Just noticeable difference (JND) value. The higher the confidence level of the response, the smaller the PSE. This means that time is perceived to be longer

Figure 6A shows the results of fitting the psychometric curves by classifying the data according to the degree of confidence in the response to visibility. The horizontal axis shows the stimulus presentation time, and the vertical axis shows the percentage of participants who answered that the presented stimulus was longer than the standard time. Figures 6B and C show the results of the JND and PSE calculations for each condition.

The analysis showed no significant difference between JND and PSE (JND: *M*_*D*_ = 0*.*05, 95% CI [*− *0*.*02*,* 0*.*13], *t*(24) = 1*.*46, *p* = *.*157, *d* = 0.29, PSE: *M*_*D*_ = 0*.*05, 95% CI [*− *0*.*03*,* 0*.*13], *t*(24) = 1*.*22, *p* = *.*236, *d* = 0.24). In other words, there was no difference in perceived time between the cases where the participants were not confident in their responses and those where they were confident in their responses.

#### Mediation analysis

Trial-level mediation analysis (*N* = 3,750 trials) revealed that VAS confidence ratings significantly mediated the effect of *g*-values on temporal responses. *g*-Values strongly predicted VAS confidence (Path a: *β* = 2.68, *p* <.001), which in turn predicted temporal responses (Path b: *β* = 1.17, *p* <.001). The indirect effect was significant (*β* = 3.14, 95% CI [1.88, 4.49], *p* <.001, log-odds scale; odds ratio = 23.1). The direct effect became non-significant when controlling for VAS confidence (Path *c'*: *p* =.185), demonstrating mediation whereby VAS confidence accounts for the relationship between stimulus properties and temporal judgments (see Online Supplementary Material Fig. S1).

#### Additional VAS analyses

To further examine the relationship between confidence judgments and temporal perception, we conducted three additional analyses.

We compared trials with high confidence in object recognition (top quartile of VAS scores) versus high confidence that no object was present (bottom quartile of VAS scores) using a generalized linear mixed-effects model. The analysis revealed a significant main effect of duration (β = 8.30, *z* = 13.28, *p* <.001), indicating that longer stimulus durations increased the probability of “Long” responses. However, the interaction between confidence category and duration was not significant (β = −0.90, *z* = −1.45, *p* =.148), suggesting that the effect of extreme confidence levels on temporal judgments was relatively consistent across different stimulus durations.

At the middle duration (1.0 s), where temporal uncertainty is maximal, we examined whether VAS scores predicted Short versus Long responses. The analysis revealed a significant positive association (β = 0.77, *z* = 3.52, *p* <.001), indicating that higher confidence in object recognition was associated with longer perceived durations even at maximum temporal uncertainty.

Previous temporal responses did not significantly influence subsequent VAS ratings (β = 0.008, *t* = 0.68, *p* =.497), and previous VAS scores did not significantly influence subsequent temporal judgments (β = −0.14, *z* = −1.13, *p* =.261). These null findings suggest minimal carryover effects between trials.

## Discussion

Our experimental manipulation successfully modulated object recognition through the *g* parameter, with higher *g* values consistently producing higher rates of object recognition (Fig. 3). This validation is crucial as it confirms that our stimuli created the intended variation in stimulus clarity while maintaining physical characteristics such as luminance and contrast.

The systematic relationship we observed between *g* values and PSE, with higher *g* values associated with lower PSE values, demonstrates that increased object visibility leads to longer perceived durations. This finding aligns with predictions from information processing accounts of time perception (Eagleman & Pariyadath, 2009), which propose that stimuli requiring more extensive neural processing are experienced as lasting longer. The specific effect of clearer object visibility (*g* = 0.30) compared to less clear stimuli (*g* = 0.05, 0.10) suggests a potential threshold effect, where duration dilation may occur primarily when object recognition exceeds a certain level of clarity.

Importantly, this modulation affected only the bias in temporal perception (PSE) without significantly altering the precision of temporal discrimination (JND). This dissociation, a shift in PSE without changes in JND, has been observed in previous timing studies (Grondin, 2010), and suggests that object recognition affects the accumulation of temporal information rather than the noise in the temporal processing system. Such a pattern indicates that our experimental manipulation influences the subjective magnitude of experienced duration without compromising the underlying timing mechanisms themselves.

The magnitude of the temporal distortion we observed (100 ms, or approximately 10% of stimulus duration) is comparable to effects reported in other studies examining attention and stimulus salience in time perception. For example, Tse et al. (2004) found approximately 20% duration expansion for oddball stimuli in visual sequences, and Matthews (2011) reported similar magnitudes for stimulus repetition effects. This consistency across studies suggests that our findings reflect a general principle: that subjectively salient or meaningful stimuli – whether defined by novelty, unexpectedness, or, as in our case, recognizability – are experienced as lasting longer. From a functional perspective, this temporal dilation may serve to allocate additional processing time to perceptually significant events, potentially facilitating more thorough cognitive processing and adaptive behavioral responses. However, as we discuss in the *General discussion*, the interpretation of these effects within processing-based frameworks requires careful consideration, as such accounts face inherent challenges regarding post hoc flexibility.

These findings raise an important question: Is the modulation of perceived time driven by the mere detection of an object, or does it require deeper processing, such as object identification and naming? To address this question, we conducted Experiment 2, which explicitly distinguished between different levels of object recognition.

## Experiment 2

### Materials and methods

#### Participants

Twenty students (18 male and two female participants; mean age 20.9 years, standard deviation 1.7 years) participated in the experiment. Similar to Experiment 1, we did not conduct an a priori power analysis for this experiment. Therefore, we conducted a sensitivity power analysis (Lakens, 2022). This analysis revealed that our design was capable of detecting effect sizes of Cohen’s *d* = 0.66, given an alpha level of 0.05 and power of 0.80, demonstrating adequate statistical power for detecting meaningful effects. All participants received a thorough explanation of the experiment and task and subsequently agreed to participate. This experiment was conducted with the approval of the “Experiments on Human Subjects” Review Committee of the Safety and Health Committee of the Toyohashi University of Technology.

#### Stimuli

Stimuli were created using the Dots method (Moca et al., 2011), following the same general principles as in Experiment 1. However, to eliminate potential habituation effects, we used a new set of images, as some participants from Experiment 1 might participate in this experiment too. The parameter *g* was set to four levels (0.05, 0.10, 0.15, and 0.30), with D and K values maintained at 5 and 10, respectively, as in Experiment 1. Original images were obtained from Caltech 101 (Fei-Fei et al., 2004), Caltech 256 (Griffin et al., 2007), Material Dictionary (DataCraft), PhotoAC (AC Works, Inc.), Japaclip (https://japaclip.com/), and Public Domain Q (https://publicdomainq.net/), converted to grayscale, resized to 600 × 400 pixels, and processed using the Dots method. An important methodological difference from Experiment 1 was the creation of mirror-image versions of each stimulus pattern to reduce the effect of repeated presentation. For example, if a stimulus contained an object on the left side, we created an additional version with the same object on the right side by horizontally flipping the image. This manipulation helped ensure that recognition performance was not influenced by repeated exposure to identical patterns.

#### Procedure

This experiment was conducted in the same experimental environment as Experiment 1. As in Experiment 1, the participants engaged in the generalization phase by memorizing the shortest and longest durations as a reference. Next, in the bisection phase, the participants performed a bisection task in which they responded to whether the presented duration of the stimuli was closer to the shortest or longest duration that they remembered in the generalization phase.

##### Generalization phase

**Fig. 7:**
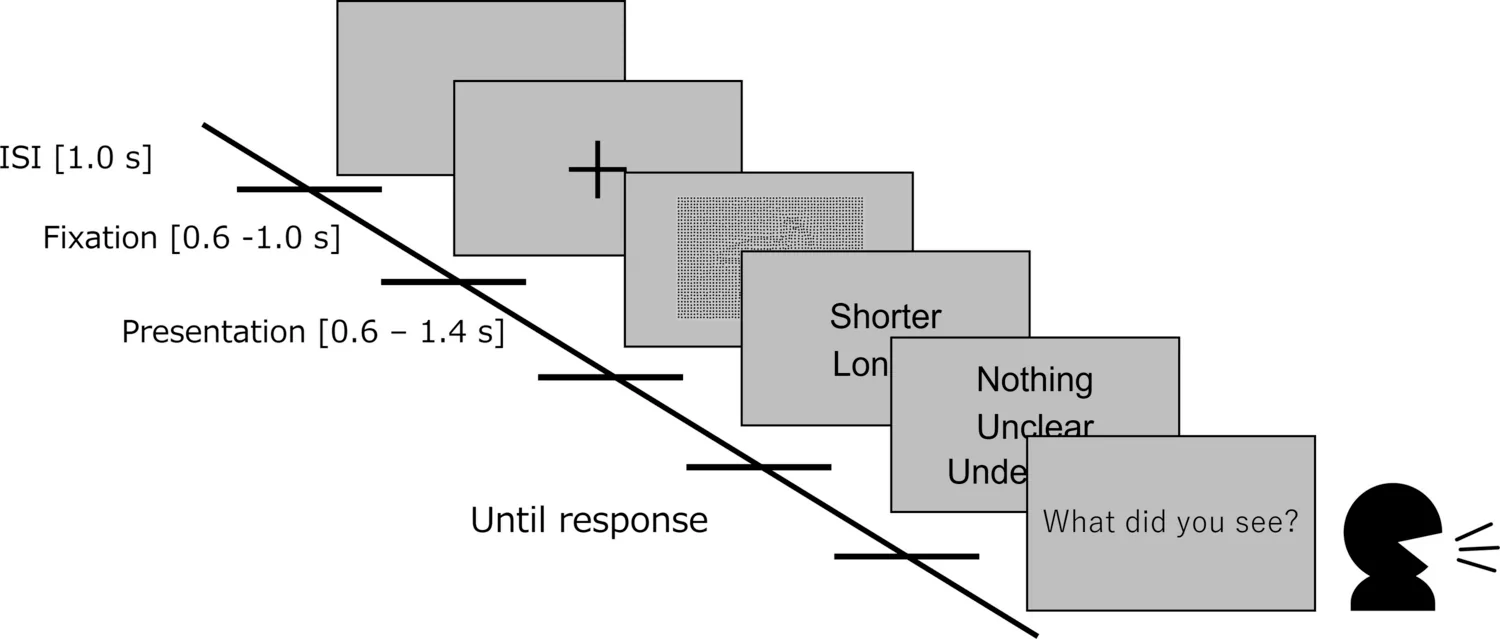
Experimental protocol for one trial in Experiment 2. Time course of a single trial. The trial begins with an inter-stimulus interval (ISI; 1.0 s), followed by a fixation cross (0.6–1.0 s), and then the stimulus presentation (0.6–1.4 s). After the stimulus offset, participants made two responses: first indicating whether the duration was closer to the shortest or longest learned duration, and then reporting their perception of the stimulus (Nothing/Unclear/Named), followed by verbal naming when applicable

The generalization phase was designed to have the participants remember the shortest (0.6 s) and longest (1.4 s) durations. Figure 7 depicts the flow of one trial.

When the task began, only the background was presented as an inter-stimulus interval (ISI) for 1.0 s, and the fixation cross was then presented randomly at 0.6, 0.8, or 1.0 s. The presentation duration of the fixation cross was randomized to avoid serving as a cue for temporal duration estimation. The stimulus was then presented for a minimum duration of 0.6 s or a maximum duration of 1.4 s. Participants were then presented with the bisection stimulus. The important procedural difference in Experiment 2 was the modification of the visibility evaluation task. Instead of the binary response used in Experiment 1, participants now responded to the presented stimulus with three response options reflecting different levels of object recognition: (1) “Nothing” – No perception of any organized structure in the stimulus. (2) “Unclear” – Detection of some organized structure suggesting the presence of an object but inability to identify what the object is. (3) “Named” – Both detection of an object and successful identification/naming of it. When participants responded “Named,” they were asked to verbally state the name of the object they recognized, which was recorded for analysis. These three response options allowed us to distinguish between two important transitions in visual processing: the transition from “Nothing” to “Unclear” (reflecting the emergence of conscious object detection) and the transition from “Unclear” to “Named” (reflecting the achievement of semantic recognition).

After responding orally, the participant proceeded to the next trial by pressing a key. The shortest (0.6 s) and longest (1.4 s) presentation times were shown three times each, for a total of six presentations made in a random order. The generalization phase was terminated when the bisection task was answered correctly six times in a row; when the bisection task was answered incorrectly even once, the order of the presentation times was rearranged randomly, and the task continued.

##### Bisection phase

In the bisection phase, the results derived from the bisection task, the visibility evaluation task, and verbal estimation were recorded and stored as study data. The flow of the experiment was the same as that in Experiment 1, except that no feedback was provided. In the bisection phase, participants were randomly presented with a presentation time of 0.6, 0.8, 1.0, 1.2, or 1.4 s. In the 2AFC task, the participants answered whether they felt that the presentation time was closer to the shortest (0.6 s) or longest (1.4 s) duration that they had learned in the generalization phase. The experiment consisted of 240 trials (5 time conditions × 4 g-value conditions × 12 repetitions), divided into six sessions. Participants were allowed to take breaks between sessions, and the generalization phase was conducted at the beginning of each session. Stimuli were presented in a random order for each participant.

Data analysis followed the same procedures as Experiment 1, using the quickpsy R package for psychometric function fitting and statistical analyses. Additionally, we conducted trial-level mediation analysis to test whether visibility classifications (0 = nothing, 1 = unclear, 2 = named) mediate the effect of *g*-values on temporal responses. The same mixed-effects modeling approach as Experiment 1 was used, with visibility treated as an ordinal variable in Path a and logistic regression used for paths involving binary temporal responses (Paths b, c, and c').

### Results

**Fig. 8:**
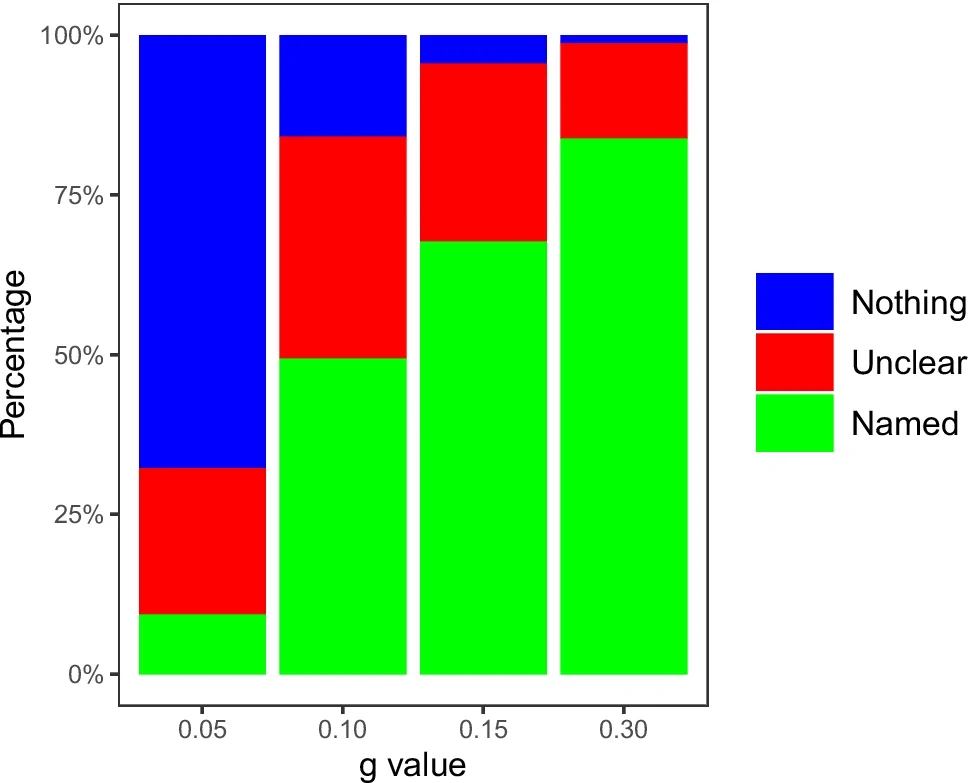
Average of the percentage of each response for each parameter *g*. It can be seen that as *g* increases, the percentage of named responses increases

Figure 8 shows that the larger the parameter *g*, the higher the percentage of responses when the object was present in the stimulus and its name was known (*χ*^2^(6*, N* = 4,800) = 2,341*.*47, *p* < *.*001).

**Fig. 9:**
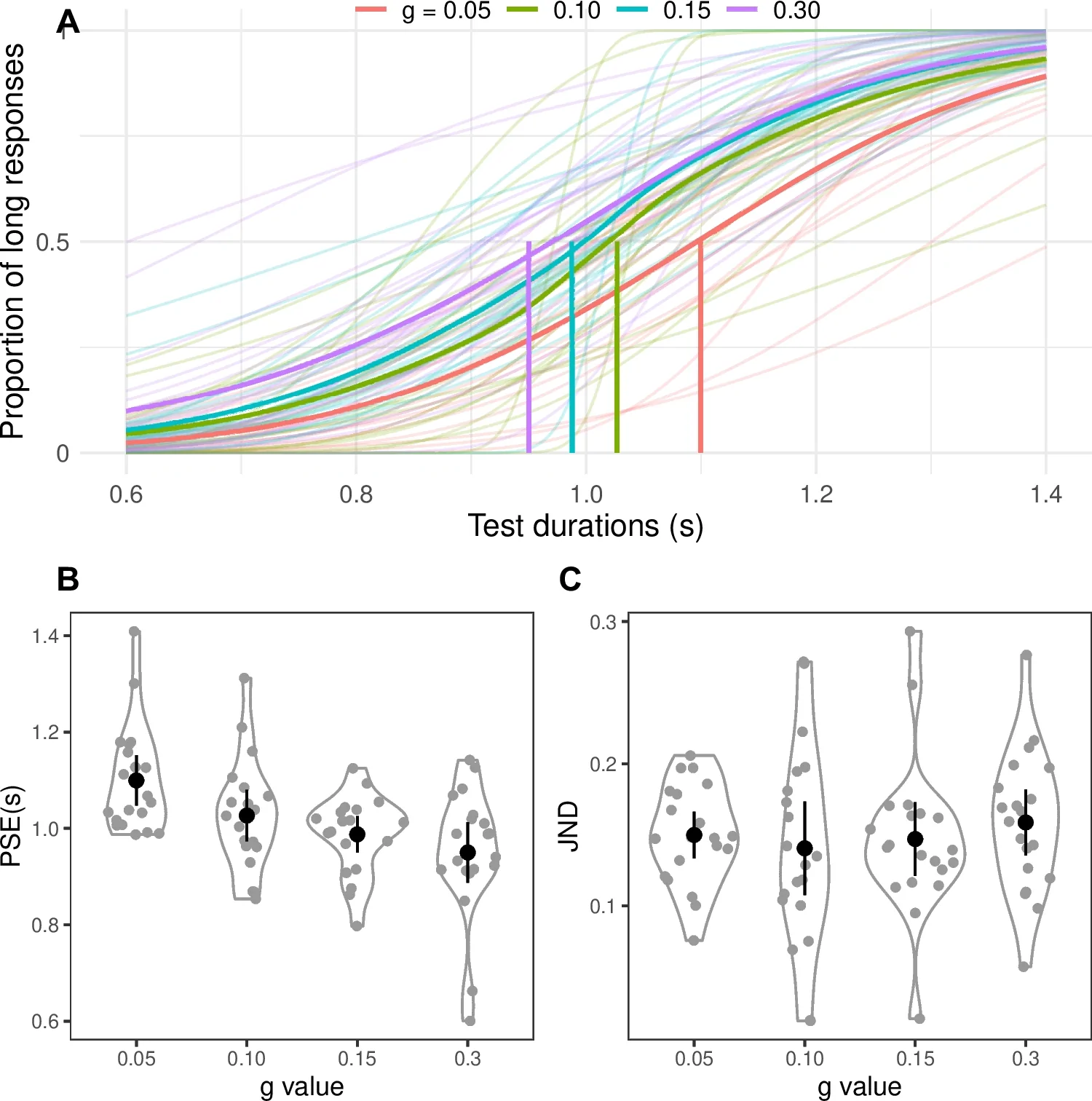
**A** Psychometric functions for each *g*. Light colors represent individuals, dark colors represent group levels, and vertical lines indicate the average point of subjective equality (PSE). **B** PSE value per *g*. **C** Just noticeable difference (JND) value per *g*. As the *g* value increases, PSE decreases, which is consistent with the results of Experiment 1

Figure 9A shows the results of fitting the psychometric curves after classifying the data according to *g*. The horizontal axis indicates the stimulus presentation time, and the vertical axis shows the percentage of responses in which the presented stimulus was close to the long duration standard. Figures 9B and C show the JND and PSE calculation results for each condition. Light colors represent individual levels, dark colors represent group levels, and vertical lines indicate the average PSE. Repeated-measures ANOVA was performed on the PSE and JND values. The factors were the four levels of parameter *g*. The analysis showed a significant main effect for PSE (*F*(1*.*94*,* 36*.*79) = 17*.*38, *MSE* = 0*.*01, *p* < *.*001, ${\widehat{\eta }}_{G}^{2}=$ *.*205), but not for JND (*F*(2*.*40*,* 45*.*66) = 0*.*92, *MSE* = 0*.*00, *p* = *.*420, ${\widehat{\eta }}_{G}^{2}$= *.*015). Significant differences were found between conditions in PSE (*g* = 0.05 and *g* = 0.10: *t*(19) = 5*.*32, *p*_Holm(6)_ < *.*001, *g* = 0.05 and *g* = 0.15: *t*(19) = 5*.*68, *p*_Holm(6)_ < *.*001, and *g* = 0.05 and *g* = 0.30: *t*(19) = 5*.*37, *p*_Holm(6)_ < *.*001). In other words, they perceived the stimuli that were easy to understand as being longer than those that were difficult to understand in terms of what the object was. Additional pairwise comparisons revealed a significant difference between *g* = 0.10 and *g* = 0.30 (*t*(19) = 2.82, *p*_*Holm(6)*_ =.033), but no significant differences between *g* = 0.10 and *g* = 0.15 (*t*(19) = 2.19, *p*_*Holm(6)*_ =.083) or between *g* = 0.15 and *g* = 0.30 (*t*(19) = 1.85, *p*_*Holm(6)*_ =.083).

To examine the nature of the relationship between response category and perceived duration, we conducted polynomial trend analyses. Results revealed a significant linear trend (*F*(1, 19) = 76.64, *p* <.001, ${\widehat{\eta }}_{G}^{2}=$.278), indicating that PSE decreased systematically as object recognizability increased from Nothing to Unclear to Named. The quadratic trend was also significant (*F*(1, 19) = 13.23, *p* =.002, ${\widehat{\eta }}_{G}^{2}=$.062), though the linear component was substantially stronger, confirming that the relationship is predominantly linear with a modest curvilinear component.

**Fig. 10:**
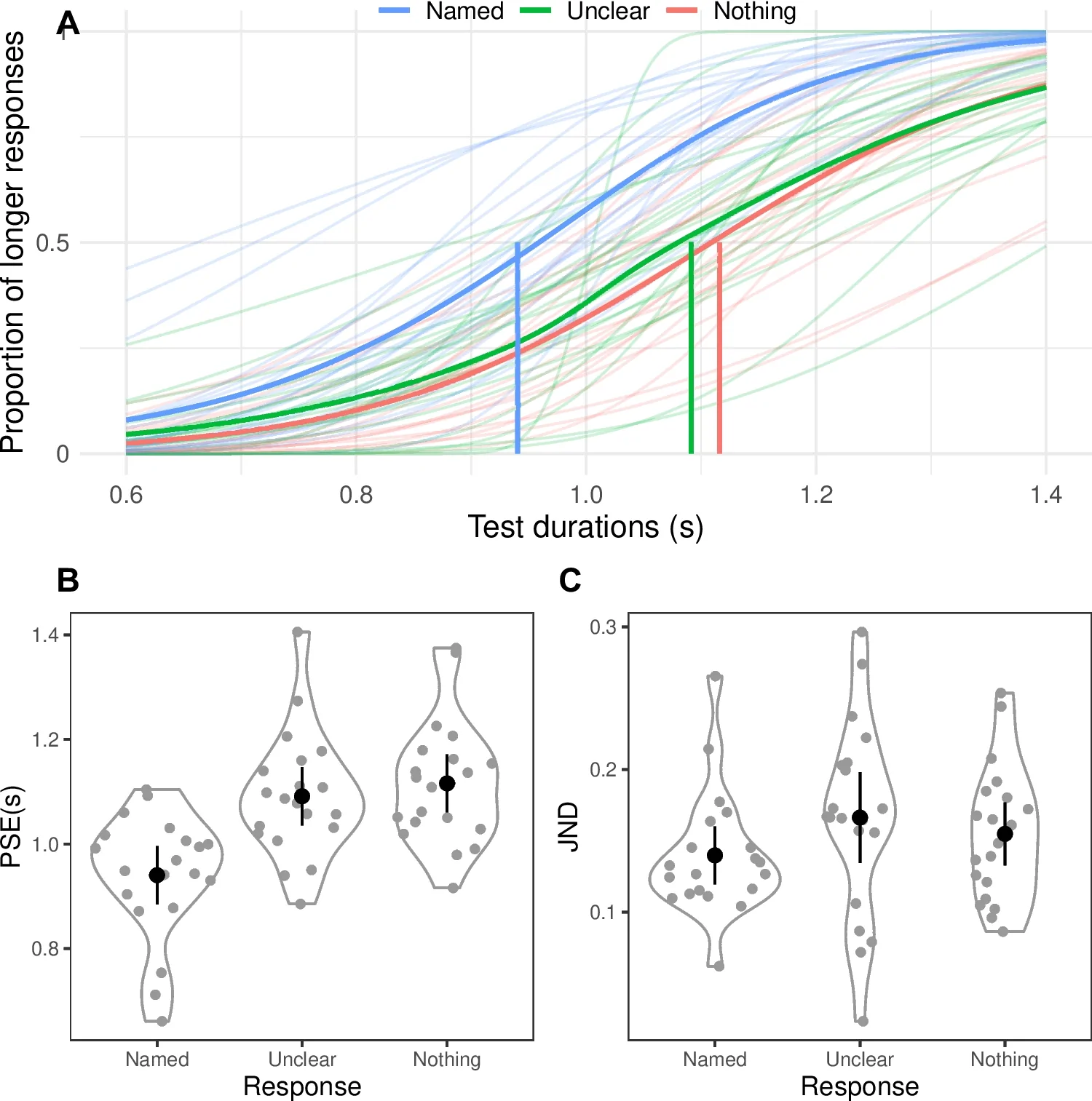
**A** Psychometric functions for each response. Light colors represent individuals, dark colors represent group levels, and vertical lines indicate the average point of subjective equality (PSE). **B** PSE value. **C** Just noticeable difference (JND) value. The PSE was smaller for those who responded that they “Named” an object in the dot stimulus. This means that time is perceived to be longer

Stimuli were classified according to the visibility response, and the results of fitting the psychometric curves are shown in Fig. 10A. The conditions were defined as follows:

“Nothing,” wherein participants responded that the object was not present or was not an object; “Unclear,” in which participants responded that the object was present but the name was not known; and “Named,” wherein participants responded that the object’s name was known. The horizontal axis shows the stimulus presentation time, and the vertical axis shows the percentage of responses for which the presented stimulus was close to the long duration standard. JND and PSE were calculated for each condition from these results and are shown in Figs. 10B and C. Light colors represent individual levels, dark colors represent group levels, and vertical lines indicate average PSE. Repeated-measures ANOVA was performed on the PSE and JND values. The factors constituted three levels of visibility. The analysis showed a significant main effect for PSE (F(1.48,28.10)=44.94, p<.001, ${\widehat{\eta }}_{G}^{2}=.311$), not for JND (F(1.82,34.61)=2.30, p=.120, ${\widehat{\eta }}_{G}^{2}=.041$). Significant differences were found between conditions in PSE (Named and Nothing: *t*(19) = 8*.*08, *p*_Holm(3)_ < *.*001, and Named and Unclear: *t*(19) = 6*.*35, *p*_Holm(3)_ < *.*001). In other words, it was found that the subjects perceived the stimuli that were easy to understand as being presented for a longer period of time than those that were difficult to understand regarding what the object was. Importantly, the difference between Nothing and Unclear conditions was not significant (*t*(19) = 1.89, *p*_*Holm(3)*_ =.074), indicating that mere object detection without successful naming did not significantly extend perceived duration.

#### Comparison with objective midpoint

To assess whether PSE values represent genuine temporal over- or underestimation, we compared each condition’s PSE with the geometric mean of anchor durations (0.917 s). For *g*-value conditions, only *g* = 0.30 showed veridical time perception (*M* = 0.950, *t*(19) = 1.13, *p* =.274, *d* = 0.25), while all other conditions showed significant temporal underestimation (*g* = 0.05: *M* = 1.100, *t*(19) = 7.36, *p* <.001, *d* = 1.64; *g* = 0.10: *M* = 1.027, *t*(19) = 4.31, *p* <.001, *d* = 0.96; *g* = 0.15: *M* = 0.988, *t*(19) = 3.96, *p* =.001, *d* = 0.89). Similarly, for response categories, only the Named condition showed veridical perception (*M* = 0.940, *t*(19) = 0.90, *p* =.378, *d* = 0.20), whereas Nothing (*M* = 1.116, *t*(19) = 7.53, *p* <.001, *d* = 1.68) and Unclear (*M* = 1.091, *t*(19) = 6.60, *p* <.001, *d* = 1.48) conditions showed significant underestimation. These results suggest that successful object recognition restores accurate temporal perception.

#### Mediation analysis

Trial-level mediation analysis (*N* = 4,560 trials) showed that visibility classifications significantly mediated the effect of *g*-values on temporal responses. *g*-Values strongly predicted visibility (Path a: *β* = 4.76, *p* <.001), which predicted temporal responses (Path b: *β* = 0.60, *p* <.001). The indirect effect was significant (*β* = 2.87, 95% CI [2.13, 3.64], *p* <.001, log-odds scale; odds ratio = 17.6). The direct effect became non-significant when controlling for visibility (Path *c’*: *p* =.076), demonstrating mediation whereby subjective recognition accounts for the relationship between stimulus properties and temporal judgments (see Online Supplementary Material Fig. S1).

## Discussion

Figure 8 shows that when the parameter *g* was set to a medium level, the number of responses in which the participants could perceive the object but could not recognize its name increased, and as *g* increased, the percentage of responses in which the participants recognized the object’s name increased. This result confirms that our manipulation of the *g* parameter successfully modulated participants’ subjective perception of the objects. The significant PSE shift from *g* = 0.05 to *g* = 0.10 is noteworthy because recognition rates at *g* = 0.10 remain relatively low. This shift may reflect the engagement of object identification processes even when naming is ultimately unsuccessful. When participants actively attempt to identify objects, rather than passively viewing dot patterns, additional processing resources may be allocated, contributing to temporal dilation independent of recognition success.

The results of Experiment 2 extend our findings by distinguishing between different levels of object recognition. Specifically, PSE values were significantly lower, indicating longer perceived durations when participants could name the perceived object compared to when they merely detected an object without naming it or saw no object at all (Fig. 10). Critically, mere object detection without naming did not produce significantly longer perceived durations compared to no object detection. This pattern suggests that semantic processing, rather than simple perceptual detection, may be an important factor in extending perceived duration.

## General discussion

Our study investigated how subjective visual perception, particularly object recognition and naming, modulates perceived duration. Across two experiments, we found consistent evidence that subjective perceptual experiences significantly influence time perception. Here, we integrate these findings and discuss their theoretical implications. In Experiment 1, we demonstrated that stimuli with higher visibility (*g* = 0.30) were perceived as lasting longer than less visible stimuli (*g* = 0.05, 0.10). When we classified the data based on participants’ subjective reports of object visibility, stimuli in which participants detected an object were perceived as lasting longer than those in which no object was detected. Experiment 2 revealed a critical distinction: it was not merely the detection of an object that extended perceived duration but specifically the ability to recognize and name the object. Stimuli for which participants could name the perceived object were judged as lasting longer than those that were either not perceived as containing an object or contained an unnameable object. Across both experiments, these effects were observed consistently in PSE values without corresponding changes in JND, indicating that object recognition affects the subjective magnitude of duration rather than the precision of temporal discrimination.

### Information processing and attention

Our findings can be interpreted within the coding efficiency framework (Eagleman & Pariyadath, 2009), which proposes that perceived duration depends on the neural resources required for stimulus processing. Recognizable objects, particularly those that can be named, likely engage more extensive neural processing – including semantic networks – than unrecognizable patterns. According to this framework, such increased neural activity may translate to extended perceived duration. However, as noted in the *Discussion* in Experiment 1, processing-based accounts face inherent interpretive challenges, as they can potentially accommodate various patterns of results. Our use of subjective measures (VAS ratings and naming ability) helps constrain these interpretations by demonstrating that subjective recognition success, rather than objective stimulus difficulty alone, predicts perceived duration.

The systematic relationship between stimulus visibility (*g*-value) and subjective time aligns with the idea that clearer object recognition mobilizes more neural resources, thereby extending perceived duration. Importantly, control analyses confirmed that objective presentation duration had minimal influence on visibility ratings (Experiment 1: *r* =.02, *p* =.18) or naming probability (Experiment 2: *r* =.06, accounting for less than 1% of variance), ruling out the possibility that longer durations simply facilitated recognition. This further supports our conclusion that subjective recognition success, rather than physical stimulus duration, is the primary driver of the observed temporal effects. In Experiment 2, this relationship was further refined, demonstrating that semantic processing (naming) rather than mere object detection plays a crucial role in extending perceived duration. This pattern is consistent with models that link subjective duration to neural energy expenditure (Eagleman & Pariyadath, 2009), suggesting that the increased metabolic demands of semantic processing may be an important factor in temporal dilation. The specific effect of naming beyond mere detection further suggests that temporal perception may be particularly sensitive to higher-order cognitive processing rather than basic perceptual operations.

### Attention allocation

The pacemaker-accumulator model provides another framework for understanding our results. This model proposes that attention acts as a gate controlling the flow of pulses from a pacemaker to an accumulator, as demonstrated in previous timing research (Brown, 1997).

Our finding that named objects are perceived as lasting longer than unnamed objects may seem counterintuitive within this framework, as naming involves non-temporal processing that should divert attention away from temporal aspects. According to the standard interpretation of the pacemaker-accumulator model, we might expect that the additional cognitive processing required for naming would reduce attention to timing, resulting in shorter perceived durations. However, this apparent contradiction can be resolved by considering that successfully named objects may actually require less attentional resources for continued processing compared to unrecognized objects that resist identification, thereby allowing more attentional resources to be allocated to temporal processing.

This interpretation is consistent with previous research showing that completing a secondary cognitive task can paradoxically extend perceived duration when it reduces uncertainty and attentional demands (Brown, 1997). Named objects, having reached a state of perceptual and semantic resolution, may free attentional resources that can then be redirected to temporal processing. In contrast, unrecognized objects that cannot be named may continue to engage attentional resources in an ongoing attempt to resolve their identity, leaving fewer resources available for temporal processing.

We note that while planned contrasts based on specific directional hypotheses would have been theoretically justified given our predictions about the effects of object recognizability, we opted for a comprehensive exploratory approach using repeated-measures ANOVA to examine all pairwise differences. The polynomial trend analyses conducted for Experiment 2 confirmed that the relationship between object recognizability and perceived duration is predominantly linear, consistent with our hypothesis. Future studies with more focused research questions may benefit from a priori contrast testing to maximize statistical power for specific theoretical predictions.

### Scalar Expectancy Theory (SET) model interpretation

To provide a more specific mechanistic interpretation, we analyzed our PSE and JND findings within the Scalar Expectancy Theory (SET) framework (Gibbon, 1977; Gibbon et al., 1984). SET proposes that temporal perception involves three stages: a clock stage (where a pacemaker generates pulses that are accumulated), a memory stage (where reference durations are stored), and a decision stage (where current duration is compared to stored standards). Changes in PSE without corresponding JND changes suggest alterations in the clock component (pacemaker rate), whereas JND changes would indicate modifications in memory variance or decision noise.

We calculated Weber Fractions (WF = JND/PSE) and relative clock rates for each condition across both experiments. In Experiment 1, the relative clock rate for the high-visibility condition (*g* = 0.30) was 1.06 compared to 0.97 for the low-visibility condition (*g* = 0.05), a 9% increase. When stimuli were classified by visibility responses, the Seen condition showed a relative clock rate of 1.05 compared to 0.95 for the Unseen condition, a 10% increase.

In Experiment 2, the effect was more pronounced. The Named condition showed a relative clock rate of 1.12 compared to 0.94 for the Nothing condition – a 19% increase. The PSE difference between the Named and Nothing conditions was substantial (0.18 s, 16.1%), while the JND difference was minimal (0.01 s, 6.7%), confirming that the dissociation reflects clock component alterations rather than changes in memory or decision processes.

This progressive increase in clock rate effects from basic visibility (10%) to semantic naming (19%) is consistent with the coding efficiency framework (Eagleman & Pariyadath, 2009): deeper levels of cognitive processing during object recognition may translate to faster pacemaker activity, leading to more pulses being accumulated and consequently longer perceived duration. Although the omnibus ANOVA for JND reached significance in Experiment 1 (*p* =.038), the effect size was small (*η*^*2*^ =.042) and no pairwise comparisons were significant, indicating that JND differences were minimal. This pattern, combined with the robust PSE effects, supports the conclusion that object recognition primarily modulates the clock stage of temporal processing, with minimal impact on memory storage or decision criteria.

### Subjective recognition and time perception

One of the important contributions of our study is demonstrating that subjective perceptual experiences, and not just physical stimulus characteristics (such as objective measures of complexity used in previous studies), influence time perception. That is, the same physical stimulus (identical *g*-value) can be perceived as having different temporal durations depending on how it is subjectively recognized.

This finding helps reconcile conflicting results from previous studies such as Sasaki and Yamada (2017), who found an effect of stimulus complexity on perceived duration using paintings, and Palumbo et al. (2014), who failed to find such an effect using abstract dot patterns. Our results support Palumbo et al.’s (2014) proposal that “complexity alters subjective duration only when visual complexity has semantic content and engages participants in associative and cognitive processes.” Specifically, our findings demonstrate that abstract patterns with identical physical complexity (same *g*-value) produced different temporal percepts only when they engaged semantic recognition processes, that is, when participants could name the perceived object. When stimuli lacked semantic content (could not be named or recognized as objects), no temporal elongation occurred despite similar levels of visual complexity. This pattern directly supports the distinction between semantic and non-semantic visual complexity: it is not physical complexity or even subjective recognizability per se, but rather the engagement of semantic and associative cognitive processes that drives temporal distortions. The distinction between objective stimulus properties and their semantic interpretability may explain why previous studies have yielded inconsistent results regarding the relationship between stimulus complexity and perceived duration.

These findings are further supported by additional analyses examining the relationship between VAS scores and temporal judgments. Notably, the positive association between confidence and perceived duration was maintained even at the point of maximum temporal uncertainty (1.0-s duration), suggesting that the effect operates independently of temporal discriminability. Moreover, the absence of carryover effects between consecutive trials confirms that our results reflect genuine perceptual changes rather than strategic response patterns or sequential dependencies.

However, we acknowledge an important limitation of processing-based explanations: their potential for post hoc flexibility. Notably, both our observed pattern (recognized objects → longer duration) and the opposite pattern (unrecognized objects → longer duration) could be accommodated within processing frameworks through different reasoning about attentional allocation and effort. While our use of subjective measures partially constrains these interpretations by demonstrating that recognition success, not objective difficulty, predicts duration, this approach does not fully resolve the interpretive flexibility inherent in processing-based accounts. Future studies employing more direct measures of processing effort (e.g., pupillometry, neuroimaging) alongside subjective reports could help distinguish between alternative explanations and reduce interpretive flexibility.

The trial-level mediation analyses, controlling for stimulus duration, provide strong evidence that subjective perceptual processing mediates the effects of stimulus properties on temporal perception. In both experiments, the direct effect of *g*-values on temporal responses became non-significant when subjective recognition measures were included as mediators. These findings support processing-based accounts wherein cognitive resources devoted to object recognition extend subjective duration (Eagleman & Pariyadath, 2009), and align with our finding that only semantically recognized objects significantly extended perceived duration.

### Levels of processing and temporal perception

The hierarchical effect observed in Experiment 2, where naming extended perceived duration more than mere detection, aligns with levels-of-processing accounts of cognition, which suggest that deeper semantic processing engages more extensive cognitive resources than shallow perceptual processing.

This connection between processing depth and temporal perception suggests that the subjective experience of time may be intricately linked to the depth and quality of cognitive processing rather than simply to the quantity of information presented. From an evolutionary perspective, this relationship may be adaptive, as events that engage deeper processing, potentially signaling greater biological significance, may benefit from being experienced as lasting longer, allowing more thorough cognitive processing and more effective behavioral responses.

## Limitations

The use of convenience samples also limits the generalizability of our findings, as our participant pool consisted primarily of university students with a strong gender imbalance (predominantly male participants). This sampling bias should be considered when interpreting the results. Future studies could also examine additional measures of temporal uncertainty, such as the point of maximal uncertainty (PMU), to provide complementary information about the psychometric function slope.

It is important to note that the act of naming itself, independent of the depth of processing required to resolve stimulus clarity, could contribute to temporal dilation. An ideal experimental control would involve perfectly clear images presented under both naming and non-naming conditions to isolate the pure effect of naming from processing-related effects. While our current design cannot fully separate these mechanisms, the systematic relationship between stimulus clarity and naming success suggests that semantic processing demands, rather than naming per se, likely drive the observed temporal effects. Future studies employing clear stimuli with explicit naming instructions would help clarify the relative contributions of naming demands versus perceptual processing to temporal perception.

While our SET analysis using Weber Fractions and relative clock rates provided evidence for clock component alterations, more formal SET model fitting explicitly estimating individual-level parameters for pacemaker rate, memory variance, and decision threshold would provide stronger mechanistic evidence. Future research employing such modeling approaches would allow more definitive distinctions between changes in different SET components.

An additional methodological limitation concerns our data collection procedure in Experiment 2. We only requested naming responses when participants indicated they could name the object (the “Named” condition), but not when they selected “Unclear” or “Nothing.” While this approach avoided participant frustration when objects were not clearly recognizable, it prevented us from verifying recognition accuracy in the “Unclear” condition. Future studies should collect naming attempts across all response categories, regardless of participants’ initial confidence. This would enable more fine-grained analyses of how recognition accuracy and subjective confidence jointly influence time perception, and would help distinguish between genuine recognition failures and low-confidence correct recognitions.

## Conclusion

In the present study, we investigated whether subjective ratings of visibility of presented stimuli modulate perception time. In Experiment 1, we confirmed that the perceived time was longer when an object was perceived than when no object was perceived in the presented stimulus. In Experiment 2, the participants engaged in a 3AFC task, answering whether or not they could perceive any object in the stimulus and name it. The results showed that perceived time was longer when the object’s name was answered than when it was not. These results indicate that even if the objective measures are almost the same, perceived time is longer when a participant perceives an object in the stimulus and knows its name. In other words, the results suggest that subjective stimulus perception modulates the perceived time.

## Supplementary Information

Below is the link to the electronic supplementary material.

Supplementary file1 (DOCX 29 kb)

## Data Availability

The datasets generated during the current study are available from the corresponding author on reasonable request.

## Code availability

The codes generated during the current study are available from the corresponding author on reasonable request.
