# Supplementary material for: Modulation of perceived time caused by stimulus clarity in object recognition
Source: Atten Percept Psychophys. 2026 Jun 16;88(6):148. doi: 10.3758/s13414-026-03295-8 (PMC13272666; doi:10.3758/s13414-026-03295-8)
Supplement: Supplementary file 1 — Supplementary file1 (DOCX 29 kb) [file 13414_2026_3295_MOESM1_ESM.docx]

**Supplementary Figure**


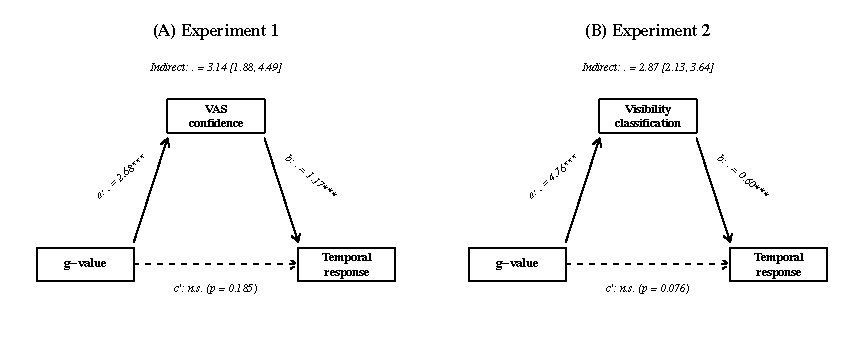


*Figure S1.* Mediation path diagrams for Experiments 1 and 2, controlling for stimulus duration. (A) Experiment 1: VAS confidence ratings completely mediated the effect of g-values on temporal responses, with the direct path becoming non-significant (dashed line). (B) Experiment 2: Visibility classifications completely mediated the effect, with the direct path also becoming non-significant (dashed line). Solid lines indicate significant paths; dashed lines indicate non-significant paths. *** *p* < .001.
